# Serum insulin-like growth factor-1 and epidemiological evidence of the risk of prostate cancer

**DOI:** 10.3389/fonc.2025.1730382

**Published:** 2026-01-09

**Authors:** Bo Fang, Hui Xiao, Ze Fang

**Affiliations:** Zhongjiang County People’s Hospital, Deyang, Sichuan, China

**Keywords:** biomarker, epidemiology, insulin-like growth factor-1, meta-analysis, prostate cancer, risk assessment

## Abstract

**Objective:**

To systematically evaluate the epidemiological association between serum insulin-like growth factor-1 (IGF-I) levels and the risk of prostate cancer, in order to provide evidence-based support for risk stratification and early prevention of prostate cancer.

**Methods:**

In accordance with the PRISMA statement, major domestic and international databases were systematically searched. Cohort studies, case-control studies, and Mendelian randomization studies reporting the relationship between serum IGF-I and prostate cancer risk were included. A random-effects model was used to combine effect estimates, assess heterogeneity, and perform subgroup analysis and meta-regression. Sensitivity analysis and publication bias tests were used to evaluate the robustness of the results, and the GRADE system was used to assess the quality of evidence.

**Results:**

A total of 16 studies involving multiple countries were included. The combined analysis showed that higher serum IGF-I levels were associated with an increased risk of prostate cancer (OR = 1.10, 95% CI: 1.02–1.18, P = 0.0136), with moderate heterogeneity (I²=50.6%). Subgroup analysis indicated that the association was more prominent in studies published in the last decade and in nested case-control designs, but heterogeneity was higher in large-sample and multicenter studies. Meta-regression analysis did not find that mean age or IGF-I levels significantly explained the heterogeneity. Sensitivity and publication bias analyses both supported the robustness of the main conclusion, and the GRADE assessment indicated moderate quality of evidence.

**Conclusion:**

Higher serum IGF-I levels are epidemiologically associated with an increased risk of prostate cancer, but the dose-response relationship is still unclear, and the correlation is susceptible to study characteristics and confounding factors. IGF-I is expected to be a potential biomarker for prostate cancer risk stratification. It is recommended that more high-quality studies be conducted in the future to verify its clinical application value.

**Systematic Review Registration:**

https://www.crd.york.ac.uk/prospero/, identifier CRD420251174259.

## 1 Introduction

### 1.1 Background

Prostate cancer (Prostate cancer, PCa) is the second most common malignant tumor among men worldwide and is also one of the important causes of cancer-related death. With the aging of the population and changes in lifestyle, the incidence of prostate cancer has continued to increase globally, especially in China, where the number of new and fatal cases has shown an increasing trend year by year in recent years, seriously threatening men’s health and causing a huge social and economic burden (1, 2). Epidemiological data show that prostate cancer is more common in people aged 65 years and above, and its risk is influenced by multiple factors, including genetics, age, hormone levels, lifestyle, etc. Although prostate-specific antigen (PSA) testing has played a certain role in early screening and diagnosis, its specificity and sensitivity are limited, and there is still a need to find more effective molecular biomarkers to achieve early diagnosis and treatment and reduce the disease burden (1, 2).

Insulin-like growth factor-1 (Insulin-like growth factor-1, IGF-I) is a polypeptide hormone secreted by the liver, its structure is similar to insulin, and it plays an important regulatory role in various physiological processes such as cell proliferation, differentiation, and apoptosis. IGF-I binds to its receptor (IGF1R), activates a series of downstream signaling pathways, promotes cell growth and survival, and inhibits programmed cell death. Existing studies have shown that IGF-I is not only essential in normal development and metabolism, but also participates in the occurrence and development of tumors, including promoting tumor cell growth and metastasis through mechanisms such as regulating the cell cycle, promoting angiogenesis, and enhancing anti-apoptotic ability (2–4). In prostate tissue, high expression of IGF-I and its receptor is considered to be closely related to the occurrence and progression of prostate cancer, and some genetic variations (such as SNPs in the 3’UTR region of IGF1R) may also regulate IGF-I signaling by affecting miRNA binding, thereby changing individual susceptibility (3).

Although a large number of epidemiological and basic studies support the correlation between IGF-I and the risk of prostate cancer, there is still considerable controversy regarding the conclusions about the relationship between the two. On the one hand, differences in IGF-I level measurement methods, sample size, follow-up time, and regional differences among different study populations lead to inconsistent results; on the other hand, some previous meta-analyses had limited sample sizes and ignored the heterogeneity of genetic background and environmental exposure (2, 3, 5). Whether there is a causal relationship between IGF-I and prostate cancer or merely a correlation phenomenon still awaits higher-quality evidence. In recent years, some large-sample cohort, Mendelian randomization, and functional studies have gradually revealed the molecular mechanisms of the IGF-I signaling axis in tumorigenesis, suggesting that it may become a potential target for tumor prevention and early screening, but there are still gaps in evidence and unclear mechanisms (1, 4, 5).

### 1.2 Objective

This study, based on epidemiological evidence of serum IGF-I levels and prostate cancer risk among different populations worldwide, combined with the latest large-sample, multicenter original studies and genetic epidemiological results, explores the application value of IGF-I as a potential biomarker in early screening and risk stratification of prostate cancer. It is hoped that this study will provide a more solid evidence base for clinical prevention, individualized screening, and molecular typing of prostate cancer, and contribute theoretical foundations and practical references to reduce the disease burden and improve patient outcomes.

## 2 Methods

### 2.1 Literature search strategy

This study was conducted in strict accordance with the reporting standards for systematic reviews and meta-analyses (PRISMA statement), and systematically searched international mainstream medical databases such as PubMed, Web of Science, and Embase. There were no restrictions on the time frame or language, and all relevant literature published since the establishment of each database was included. The search strategy combined subject terms and free words, with the main keywords including “IGF-I”, “insulin-like growth factor-1”, “prostate cancer”, “risk”, etc. (see **Supplementary Table 1** for the specific search strategy). At the same time, the references of the included literature were traced to supplement any missing relevant studies. All literature retrieval, screening, and inclusion processes were independently carried out by four researchers, and any disagreements were resolved through discussion among all authors. This study has been registered on the PROSPERO platform, with the registration number (CRD420251174259).

### 2.2 Inclusion and exclusion criteria

Inclusion criteria:

1. The study subjects are adult males;
2. The study reports the correlation between serum IGF-I levels and the risk of prostate cancer, and provides extractable effect values (such as odds ratio [OR], relative risk [RR], hazard ratio [HR] and their 95% confidence intervals);
3. The study design type is cohort study or case-control study.

Exclusion criteria:

1. Literature type is review, case report, mechanistic experimental study, or animal experiment;
2. Literature that does not provide original data or from which relevant effect values cannot be extracted.

### 2.3 Data extraction

Key information from the included studies was independently extracted by four researchers, including: first author, year of publication, country or region, study design type, sample size, age of participants, follow-up duration, mean serum IGF-I, effect values and their 95% confidence intervals (OR, RR or HR and 95% CI), as well as the main adjusted (corrected) factors. If data in the literature were incomplete, the authors were contacted or the supplementary tables were consulted to obtain the required information. In case of disagreement on data, all researchers reviewed and discussed to ensure the accuracy and consistency of the data.

### 2.4 Quality assessment

The included non-randomized intervention studies were assessed for risk of bias using the internationally recognized Risk Of Bias In Non-randomized Studies of Interventions (ROBINS-I) tool, which can systematically evaluate the risk of bias in each domain of non-randomized studies. Included Mendelian randomization studies were evaluated using the Risk Of Bias in Mendelian Randomization Studies (ROB-MR) tool, which is currently the most authoritative risk assessment method for Mendelian randomization studies internationally. All quality assessments were completed independently by two researchers and reviewed by the first author; any disagreements were resolved through discussion among all researchers to ensure the objectivity and consistency of the evaluation.

### 2.5 Statistical analysis methods

All analyses in this study used a random-effects model to combine the effect estimates from the included studies. Heterogeneity among studies was assessed using the Cochrane Q test and the I² statistic. Sensitivity analysis was performed to evaluate the robustness of the results by sequentially removing each study and recombining the effect estimates. To explore potential influencing factors, subgroup analyses were conducted according to variables such as year of publication, study type, sample size, age, and serum IGF-I, and meta-regression analysis was carried out to further assess the impact of related covariates on the results. Publication bias was assessed by visual inspection of funnel plots and quantitatively evaluated using Egger’s test and Begg’s test. All data analyses were performed using R software (version 4.4.3), and literature management and deduplication were performed using Zotero 6.0.37.

## 3 Results

### 3.1 Literature screening process

**Figure 1:**
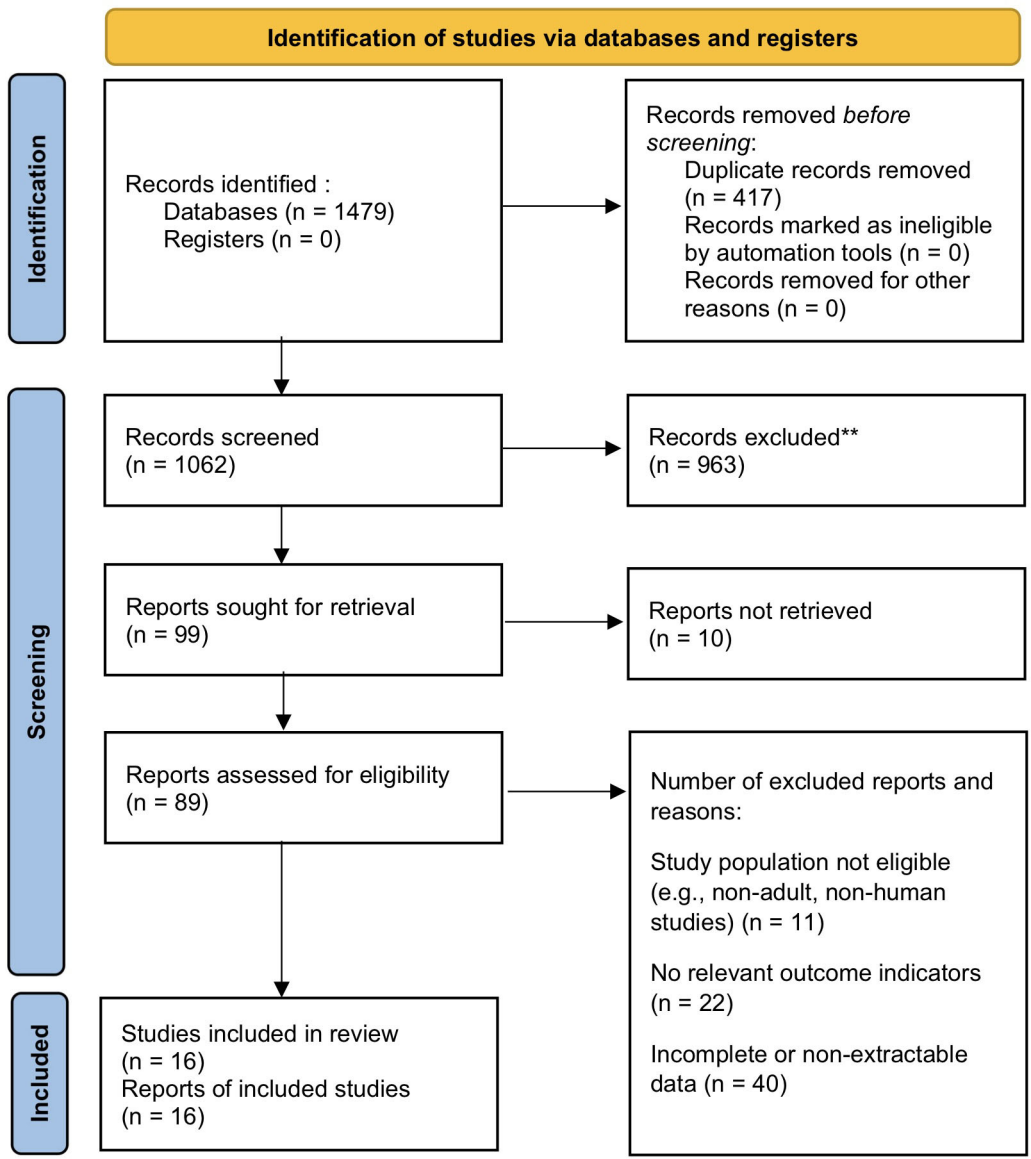
PRISMA flow diagram of literature screening.

A total of 1,479 articles were retrieved in this study, and 1,062 remained after removing duplicates. After preliminary screening of titles and abstracts, 99 articles entered the full-text review stage. Among them, 10 articles were excluded because the full text could not be obtained. The remaining 89 articles entered the eligibility assessment stage and were further excluded for the following reasons: study subjects did not meet the inclusion criteria (such as non-adults, non-human studies, etc.); relevant outcome indicators were not reported; data were incomplete or could not be extracted. A total of 16 studies met the inclusion criteria and were included in the meta-analysis. For the specific screening process and reasons for exclusion at each stage, see **Figure 1**.

### 3.2 Basic characteristics of included studies

A total of 16 studies were included, comprising prospective nested case-control studies (NCC), case-control studies (CC), population-based case-control studies (PBCC), and Mendelian randomization studies (MR). The study subjects covered multiple countries and regions including the United States, Europe, the United Kingdom, and India. The sample sizes ranged from 50 cases to over 290,000 cases, with the maximum average follow-up time up to 18 years. The ages of participants in each study were mainly concentrated in middle-aged and elderly men, and the mean serum IGF-I levels varied greatly. All studies reported effect values for serum IGF-I and prostate cancer risk, along with their 95% confidence intervals. For the specific basic information of the included studies, see **Table 1** and **Supplementary Table 2**.

**Table 1:** Summary of main results.

First author	Year	Region	Study design	Sample size	Age (years)	Follow-up (years)	Mean serum IGF-I(ng/ml)	OR (95% CI)
Li H (6)	2007	US	NCC	661	69.4 ± 7.3	18	192.6 ± 2.9	1.00 (0.76 - 1.30)
Chan JM (7)	1998	US	NCC	152	69.4 ± 7.3	7	269.4	0.98 (0.46 - 2.60)
Borugian MJ (8)	2008	US/Canada	NCC	96	67.1 ± 6.1	>1	236 ± 75	1.26 (0.66 - 2.41)
Stattin P (9)	2000	Sweden	NCC	149	67.4 ± 7.2	6.7	194 ± 62	2.37 (1.13 - 4.97)
Wolk A (10)	1998	Sweden	CC	210	50 – 74	NA	170.6	1.46 (0.82 - 2.61)
Lin Z-s (1)	2025	UK	MR	291,274	57.1 ± 7.7	10	134 ± 37	1.12 (1.04 - 1.22)
Pär Stattin (9)	2000	Sweden	NCC	149	67.5 ± 7.1	3.85	229	1.57 (0.88 - 2.81)
Mari-Anne Rowlands (11)	2012	UK	PBCC	2,686	61.9 ± 5.0	NA	163.5 ± 54.9	0.99 (0.93 - 1.04)
Afreen Khan (2)	2024	India	CC	50	70.24 ± 8.44	NA	360.47 ± 27.03	1.06 (0.72 - 1.54)
Steven E. Oliver (12)	2004	UK	PBCC	176	62.2	NA	130.7	0.81 (0.45 - 1.81)
Gu F (13)	2010	Europe	NCC	2,664	67.0 ± 4.6	9.5	191.2 ± 77.6	0.99 (0.93 - 1.05)
Fredrick R	2010	Europe	NCC	6,012	68	10	153 – 176	1.21 (1.07 - 1.36)
Tan VY (14)	2018	UK	MR	44,825	62.59 ± 5.00	NA	157.69 ± 52.61	1.14 (1.02 - 1.28)
Ma C (15)	2022	US	NCC	1,302	68.5 ± 7.1	15.7	143.0 ± 34.2	1.42 (1.04 - 1.92)
Hallmans G (16)	2004	Sweden	NCC	149	61.7 ± 6.6	2.6	156.3 ± 45.7	1.15 (0.63 - 2.09)
Mucci LA (17)	2010	US	NCC	545	68.6 ± 6.2	8.5	117.3 ± 31.7	1.09 (0.76 - 1.57)

NCC, nested case-control study; CC, case-control study; MR, Mendelian randomization study; PBCC, population-based case-control study; NA, not available.

### 3.3 Risk of bias assessment of included studies

**Figure 2:**
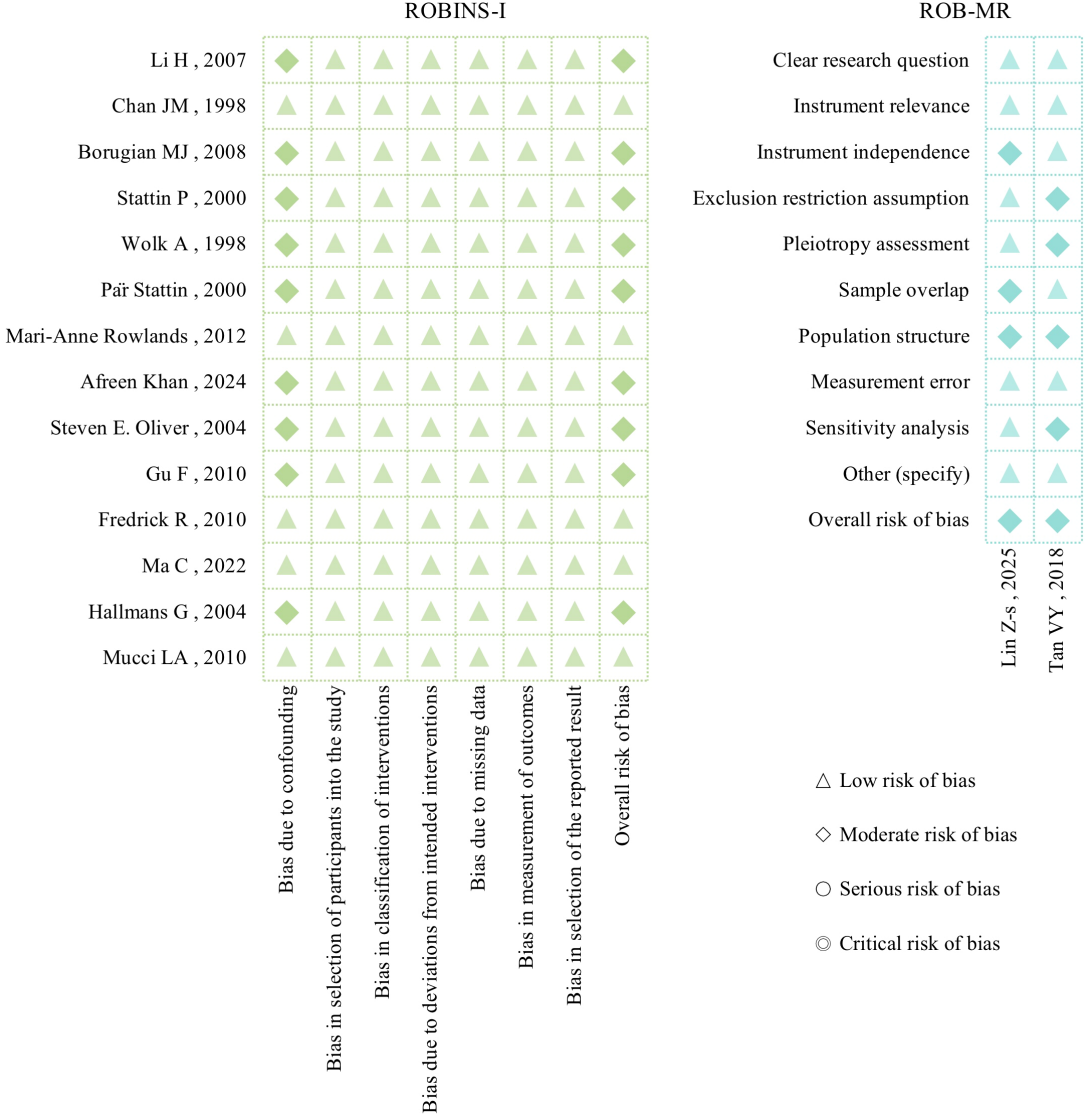
Risk of bias assessment of included studies.

The ROBINS-I tool was used to assess the risk of bias in the included non-randomized intervention studies. Most studies had a moderate risk in terms of confounding factors (Moderate risk), while the remaining domains were mostly assessed as low risk (Low risk). The overall risk of bias was mainly concentrated in the control of confounding factors. For the included Mendelian randomization studies, the ROB-MR tool was used for evaluation. The two studies were both assessed as having a moderate risk of bias (Moderate risk), mainly limited by instrument variable independence, sample overlap, and pleiotropy. For detailed evaluation results of each dimension in each study, see **Supplementary Table 3**. The results of the risk of bias assessment are shown in **Figure 2**.

### 3.4 Main effect analysis of serum IGF-I and risk of prostate cancer

**Figure 3:**
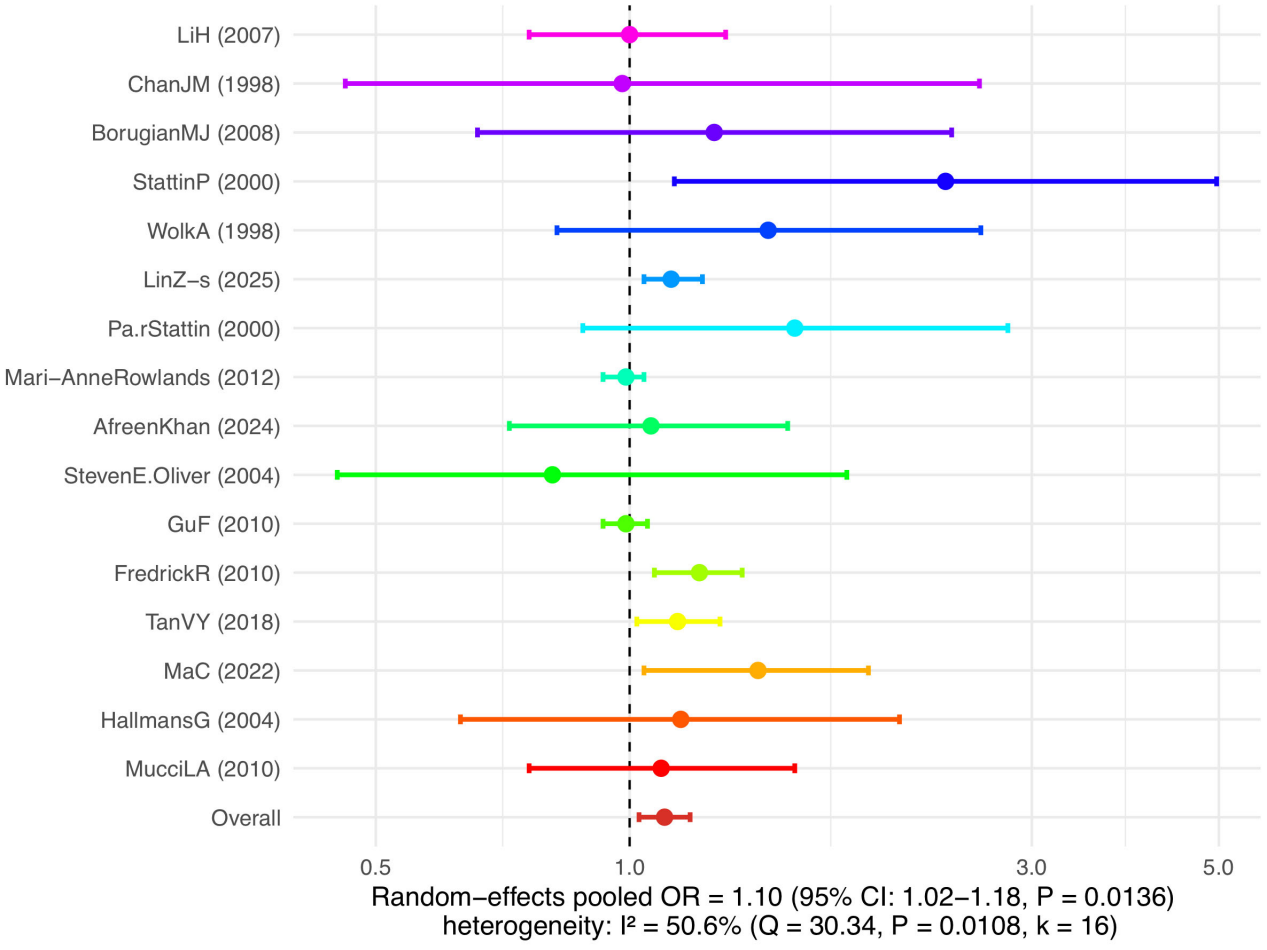
Pooled analysis results of serum IGF-I levels and risk of prostate cancer.

A random-effects model was used to combine the analysis of the relationship between serum IGF-I levels and the risk of prostate cancer. The results showed that higher serum IGF-I levels were associated with an increased risk of prostate cancer, with a combined odds ratio (OR) of 1.10 (95% CI: 1.02–1.18, P = 0.0136), which was statistically significant. The heterogeneity test result was I² = 50.6%, and the Q test P = 0.0108, indicating a moderate degree of heterogeneity among the studies. For the effect values and confidence intervals of each study, see **Figure 3**.

### 3.5 Subgroup analysis

To further explore the sources of heterogeneity between serum IGF-I and the risk of prostate cancer, this study conducted subgroup analyses on variables such as publication time, study design type, and sample size. The numerical results are presented in **Table 2**, and the corresponding subgroup forest plots are provided as **Supplementary Figures 1–6**.

**Table 2:** Subgroup analyses.

Subgroup	k	OR (95%CI)	P-value	I²	Q
Publication period					
Studies published before 2010	8	1.19 (0.94 - 1.52)	0.1305	8.30%	7.63
Studies published in or after 2010	8	1.09 (1.00 - 1.19)	0.0493	67.40%	21.44
Study design					
Nested case-control studies within cohort	10	1.15 (1.00 - 1.32)	0.0443	54.50%	19.78
Other study types	6	1.07 (0.98 - 1.18)	0.1143	52.60%	10.54
Sample size					
Sample size < 1000	10	1.13 (0.96 - 1.33)	0.1144	0.00%	7.86
Sample size ≥ 1000	6	1.09 (0.97 - 1.23)	0.1037	76.60%	21.38

1. Grouped by publication time, the combined OR of the 8 studies published before 2010 was 1.19 (95% CI: 0.94–1.52, P = 0.1305), which did not reach statistical significance, and the heterogeneity was low (I²=8.3%), indicating that the results of early studies were more consistent, but the statistical evidence was limited. The combined OR of the 8 studies published in or after 2010 was 1.09 (95% CI: 1.00–1.19, P = 0.0493), the result was statistically significant, but the heterogeneity increased significantly (I²=67.4%), suggesting that as the number and scope of studies expanded, the diversity of study population background, detection technology, and analytical methods led to greater differences in results. Therefore, although recent studies provide more evidence, the consistency of their results has declined.
2. Grouped by study design type, the combined OR of nested case-control studies within cohort (NCC, 10 studies) was 1.15 (95% CI: 1.00–1.32, P = 0.0443), indicating a statistically significant association between high IGF-I levels and increased risk of prostate cancer, and the heterogeneity was at a moderate level (I²=54.5%); while other types of studies (including case-control, Mendelian randomization, etc., 6 studies) had a combined OR of 1.07 (95% CI: 0.98–1.18, P = 0.1143), with no statistical significance, and heterogeneity was also at a moderate level (I²=52.6%). This result suggests that the NCC design may better reflect the potential causal relationship between high IGF-I and the risk of prostate cancer.
3. Grouped by sample size, the combined OR of studies with a sample size less than 1,000 (10 studies) was 1.13 (95% CI: 0.96–1.33, P = 0.1144), which did not reach statistical significance, but heterogeneity was zero (I²=0.0%), indicating that the results of these small-sample studies were highly consistent, but the inference ability was limited due to the small sample size. In contrast, studies with a sample size ≥1,000 (6 studies) had a combined OR of 1.09 (95% CI: 0.97–1.23, P = 0.1037), which also did not reach statistical significance, and heterogeneity increased significantly (I²=76.6%), reflecting that differences in population composition, measurement methods, and other aspects in large-sample, multicenter studies may increase the inconsistency of study results.

In summary, subgroup analysis found that the association between high serum IGF-I and increased risk of prostate cancer was more prominent in studies published in the past decade and in NCC designs, but heterogeneity increased with the expansion of sample size and research background. It should be noted that although the main effect analysis reached statistical significance, the combined effects of some subgroups (such as large samples, certain study types) were not statistically significant. This phenomenon is relatively common in meta-analysis, mainly due to the reduction of sample size and statistical power after subgroup splitting, as well as the increased heterogeneity brought by large-sample or multicenter studies. It is worth noting that the effect direction of the vast majority of subgroups is consistent with the main analysis, suggesting that the overall association may truly exist, but the specific effect strength and consistency still need to be further verified by larger sample size and higher quality studies. The above results indicate that, although the overall evidence supports high IGF-I as a potential risk factor for prostate cancer, different study characteristics affect the effect size and consistency, and it is still necessary to combine sensitivity analysis and meta-regression to further explain the sources of heterogeneity.

### 3.6 Meta-regression analysis

**Figure 4:**
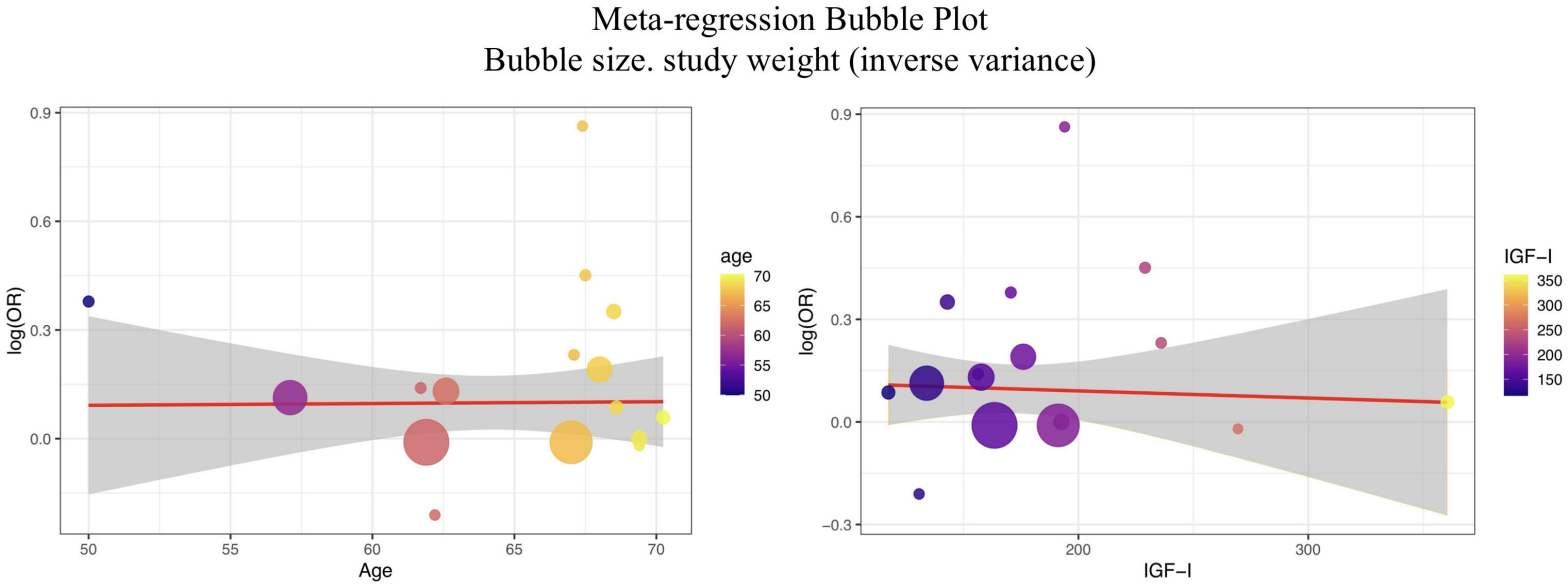
Meta-regression.

To further explore the sources of heterogeneity in the association effect between serum IGF-I and the risk of prostate cancer, this study conducted meta-regression analyses using the mean age of study subjects and the mean serum IGF-I value as covariates (**Figure 4**).

When using the mean age of study subjects as a covariate for meta-regression analysis, the results showed that the explanatory power of the age variable on the combined effect among studies was extremely limited (R²=0.00%), with a regression coefficient of 0.0005 and P = 0.9526, which was not statistically significant. The overall model test (QM = 0.0035, P = 0.9526) was also not significant, suggesting that there was no significant linear relationship between the average age of different studies and the effect size of the association between IGF-I and prostate cancer risk. In addition, the residual heterogeneity remained high (I²=54.06%, QE = 29.90, P = 0.0079), indicating that the age variable could not effectively explain most of the effect differences between studies. In other words, the variation in average age among the included studies did not statistically significantly affect the association strength between IGF-I and prostate cancer risk. Therefore, the current evidence does not support average age as a main source of heterogeneity in this study.

The meta-regression analysis with mean serum IGF-I as a covariate also showed that its explanatory power on the combined effect among studies was likewise extremely limited (R²=0.00%), with a regression coefficient of -0.0002 and P = 0.8121, which was not statistically significant. The overall model test (QM = 0.0565, P = 0.8121) was also not significant, and the residual heterogeneity remained high (I²=53.11%), further suggesting that the mean IGF-I value was not a major factor affecting the effect differences. The above results indicate that no significant linear relationship was observed between changes in mean serum IGF-I across different studies and the combined effect on prostate cancer risk.

It is important to emphasize that meta-regression can only provide an exploratory analysis of the statistical relationship between covariates and effect values, and its explanatory power and robustness are limited, especially when the number of included studies is small. Although this study attempted to include major demographic and biological indicators that might influence the effect differences, the current analysis did not find that average age or mean IGF-I had a significant explanatory effect on heterogeneity. Combined with the results of the residual heterogeneity tests, this suggests that other potential covariates (such as region, ethnicity, study design, detection method, lifestyle, etc.) may be involved in influencing the effect differences between studies.

In summary, meta-regression analysis did not find that average age or mean serum IGF-I had significant explanatory power for effect heterogeneity. The main source of heterogeneity in this study remains unclear. It is recommended that future research expand the sample size, include more possible influencing factors, and use multivariate meta-regression and stratified sensitivity analysis to more comprehensively and systematically reveal the root causes of heterogeneity in the association between serum IGF-I and prostate cancer risk, providing a more solid theoretical basis for subsequent evidence-based research and molecular epidemiological prevention and control.

### 3.7 Sensitivity analysis

**Figure 5:**
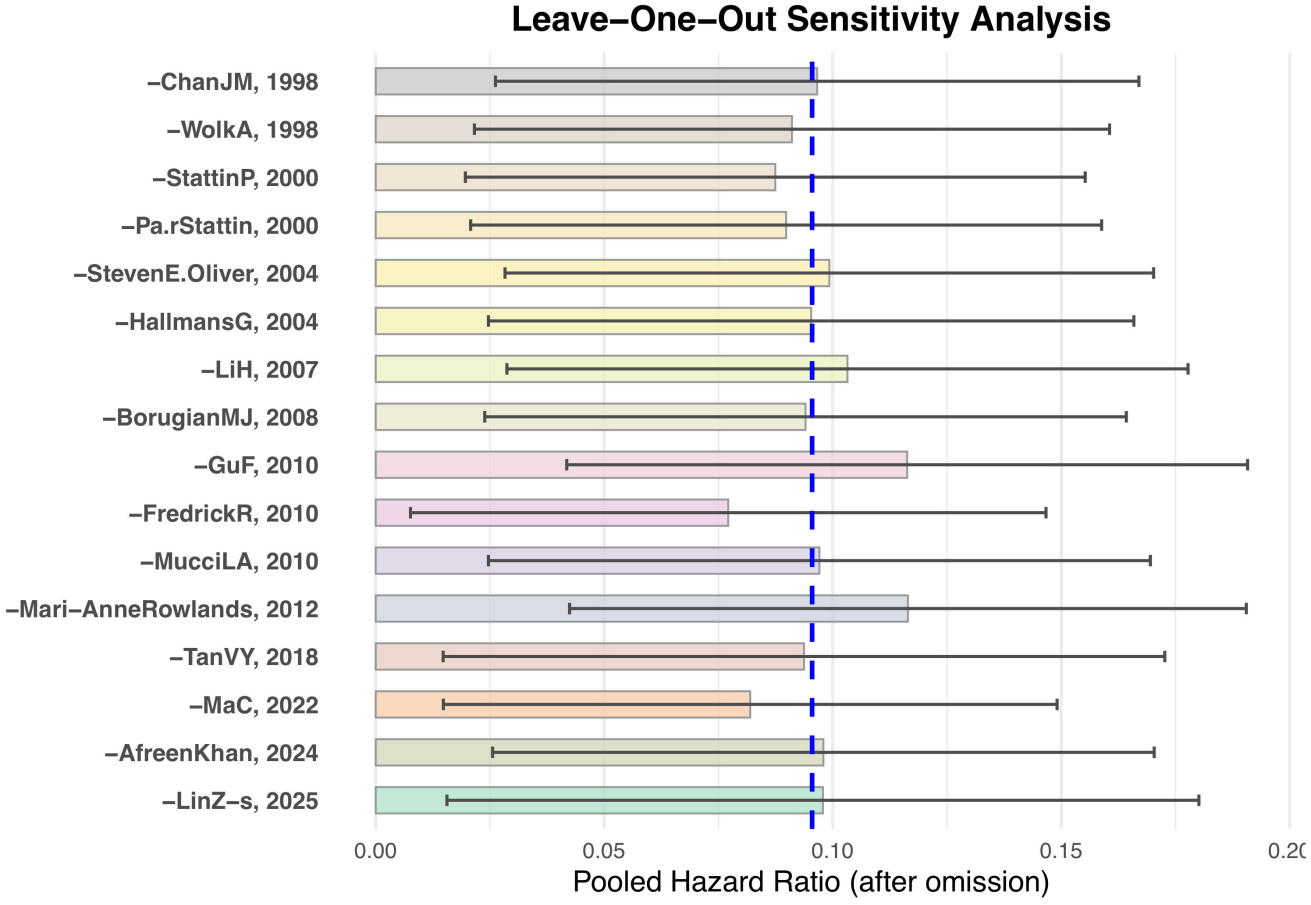
Changes in pooled effect values after leave-one-out analysis.

To evaluate the robustness of the combined results, this study performed leave-one-out sensitivity analysis on the 16 included studies. The results showed that, regardless of which study was excluded, the change in the combined effect size was minimal, and the results of the main effect analysis did not undergo substantial change (see **Figure 5**). This suggests that the results of this meta-analysis are robust and not significantly influenced by any single study.

### 3.8 Publication bias analysis

**Figure 6:**
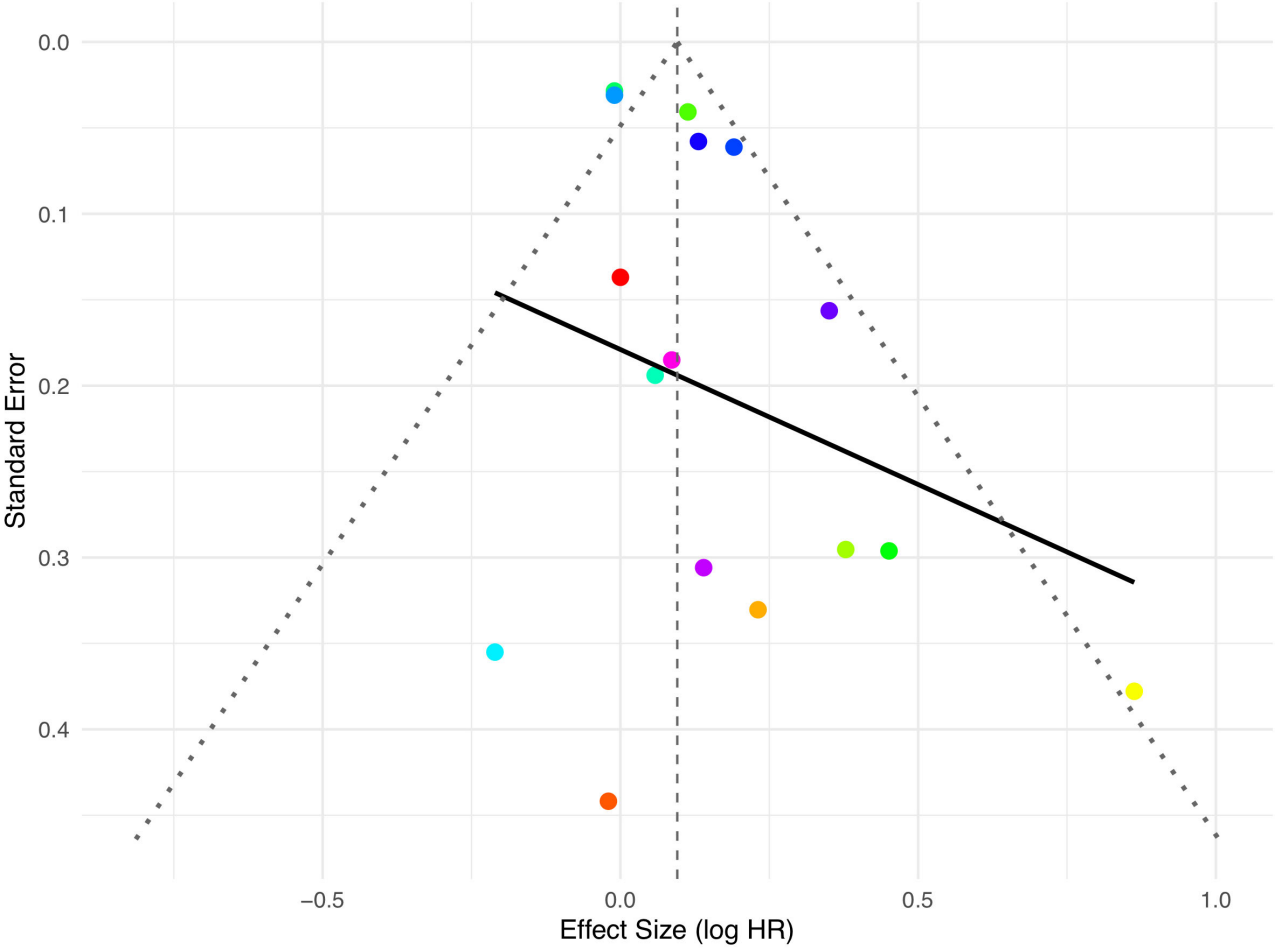
Publication bias assessment of serum IGF-I and prostate cancer risk meta-analysis.

To assess the risk of publication bias in the included studies, this study plotted a funnel plot and combined it with Egger’s test and Begg’s test for analysis (see **Figure 6**). The funnel plot showed that the distribution of study points was relatively symmetrical, with no obvious asymmetric clustering, suggesting a low risk of publication bias. The result of Egger’s test showed that the regression intercept z value was 1.8364, P = 0.0663, which did not reach statistical significance, indicating no significant bias in the funnel plot. The Begg’s test Kendall’s tau was 0.0500, P = 0.8248, also without statistical significance. This further supports that there is no obvious publication bias in the included literature as a whole. The robustness of the study conclusions is relatively good.

### 3.9 GRADE quality of evidence

This meta-analysis evaluated the quality of evidence of the included studies according to the GRADE system. Taking into account study design, risk of bias, heterogeneity, precision, and publication bias, the overall quality of evidence was rated as moderate. For the specific evaluation process and results, see **Supplementary Table 4**.

## 4 Discussion

### 4.1 Summary of main findings and comparison and interpretation with previous studies

This study systematically evaluated the relationship between serum IGF-I levels and the risk of prostate cancer. The combined analysis showed that there is a certain positive correlation between high serum IGF-I levels and prostate cancer risk, but meta-regression did not observe a clear dose-response relationship; that is, elevated IGF-I levels do not necessarily present a “gradient” phenomenon in which the risk increases linearly. Subgroup analyses also showed some discrepancies in the conclusions of studies involving different populations, detection methods, and adjustment for confounding factors. Overall, IGF-I has a certain epidemiological basis as a biomarker for prostate cancer risk, but its dose-effect relationship remains unclear.

#### 4.1.1 Supportive and opposing evidence from existing studies

Some meta-analyses and large cohort studies support the association between elevated IGF-I and increased risk of prostate cancer. The systematic review and meta-analysis by Schmitz-Dräger et al. clearly pointed out that high IGF-I levels are associated with the occurrence of prostate cancer (18). The latest Mendelian randomization analysis also found that IGF-I polymorphism has a causal relationship with the risk of prostate cancer (r=1.12, P = 0.004), suggesting that IGF-I affects the occurrence of prostate cancer at the genetic level (1). However, the gradient of this relationship is not significant: the risk increase mainly occurs in populations with higher IGF-I, and the linear relationship with its specific values is unclear. The results of meta-regression and subgroup analyses further support this point.

#### 4.1.2 Comparison with previous meta-analyses, cohort studies, and Mendelian randomization progress

Previous meta-analyses mostly used comparisons between high and low groups or quantile groups (such as highest group *vs*. lowest group), all showing a positive correlation, but results regarding whether continuous IGF-I increase (such as per 1 SD or per 10 ng/ml) continues to increase risk were inconsistent. Lin et al.’s Mendelian randomization study was the first to use genetic instrumental variables to directly infer the causality between elevated IGF-I and increased risk of prostate cancer, overcoming confounding and reverse causation in conventional observational studies, but also failed to confirm a linear trend of dose increment (1). Some cohort studies have also suggested that factors such as population (Asian *vs*. European and American), detection method, and follow-up time may all affect the strength of the association (18). With the influence of environmental exposures such as nutrition and hormones, the long-term “programming effect” of IGF-I and its relationship with tumor susceptibility have gradually attracted attention; the relationship between dairy intake and early IGF-I levels and dynamic changes in IGF-I after adulthood may all lead to differences between populations (5).

### 4.2 Possible biological mechanisms and multifactorial interpretation

#### 4.2.1 Biological basis of IGF-I in tumorigenesis and progression

Insulin-like growth factor I (IGF-I) is an important molecule for maintaining cell growth, differentiation, and apoptosis, and plays a central regulatory role in the development and progression of various tumors. IGF-I binds to its receptor (IGF-1R) and activates downstream signaling pathways such as PI3K/AKT and MAPK, promoting cell proliferation and inhibiting apoptosis, thereby driving the development and progression of solid tumors such as prostate cancer. Both epidemiological and experimental studies have confirmed that IGF-1R expression in tumor tissue is significantly higher than in normal tissue, and its high expression is closely related to the aggressiveness and lethality of prostate cancer (15). IGF-I signaling can also promote the survival and migration of tumor cells through multiple pathways, including influencing the androgen axis, DNA damage repair, and the tumor microenvironment (15).

At the fluid level, the vast majority of circulating IGF-I exists in complex form (mainly bound to IGFBP-3 and the acid-labile subunit ALS), with only 1%–5% in the free biologically active form (15, 17). Different subtypes of binding proteins (such as IGFBP-3, IGFBP-4, ALS, etc.) finely regulate the signal activity of IGF-I by modulating its stability, half-life, and accessibility. PAPP-A can hydrolyze IGFBP-4 to release free IGF-I and enhance its local biological effect; this regulatory mechanism also plays an important role in the progression of tumors such as prostate cancer (15).

#### 4.2.2 Multifactorial explanations for the lack of a dose-response relationship

Although basic and epidemiological studies support the tumor-promoting role of IGF-I in the occurrence of prostate cancer, no obvious “increasing gradient” relationship was observed in meta-regression and dose-response analysis. This phenomenon may be influenced by the following multiple factors:

1. Genetic heterogeneity and complexity of signaling networks. Large-scale genetic association studies have shown that circulating levels of IGF-I and its major binding protein IGFBP-3 are regulated by multiple genes, and common SNPs explain only a very small part (<5%) of the phenotypic variation of IGF-I/IGFBP-3. The complexity of genetic background leads to different strengths of the association between IGF-I and tumor risk in different populations (13, 19). The IGF-I signaling pathway also undergoes cross-regulation with multiple growth factors, hormones, and inflammatory molecules, making its effect not a simple “quantity change” directly driving a “quality change” (13).
2. Nonlinear effects of binding proteins and ligand-receptor regulation. The biological activity of IGF-I is regulated by a variety of binding proteins such as IGFBP-3 and ALS. Changes in their levels affect the proportion, half-life, and tissue distribution of free IGF-I. Some studies have found that elevated ALS can reduce IGF-I’s ability to cross the endothelial barrier and inhibit tissue exposure; while dynamic balance changes among binding proteins mean that an increase in total IGF-I does not necessarily equate to a continuous increase in biologically active IGF-I, thus weakening the linear feature of the dose-response relationship (15, 17).
3. Environmental factors and exposure confounding. IGF-I levels are influenced by various environmental factors such as diet (especially dairy intake), nutritional status, and exercise, and these factors vary significantly in distribution among populations (13, 19). The “early programming” effect of nutritional exposure and lifetime exposure patterns may dilute the dose-response relationship among different subgroups.
4. Detection methods and data heterogeneity. Differences in IGF-I assay methods (such as ELISA, immunochemiluminescence, etc.) and sample processing procedures among studies may result in inconsistencies in absolute values and grouping standards, increasing the heterogeneity of the dose-response curve and thereby weakening the statistical detection power (19).
5. Interaction effects of tumor subtypes and genetic background. Recent studies have shown that the influence of the IGF-I pathway is mainly concentrated in highly aggressive or lethal prostate cancer, and the effect on early, low-risk tumors is limited (15). Genotype (such as PTEN deletion status), tumor tissue IGF-1R expression level, etc., interact with the risk effect of IGF-I, further weakening the dose-response trend in the overall population.

In summary, IGF-I plays an important role in the occurrence and development of malignant tumors such as prostate cancer by activating multiple tumor-related signaling pathways, but is affected by multiple factors such as genetic background, complexity of signaling pathways, binding protein regulation, environmental exposure, detection standards, and subtype differentiation, making it difficult to present a simple linear increase in the “dose-response” relationship in the general population. Future mechanistic research should strengthen the systematic analysis of multi-component networks and their dynamic balance, and combine genetic, molecular, and environmental exposure information to clarify the true pathogenic mechanism of IGF-I in tumorigenesis.

### 4.3 Clinical and public health significance

#### 4.3.1 Prostate cancer risk warning and high-risk population identification

This study and related literature have confirmed that serum IGF-I and its related molecules (such as IGFBP-3 and pregnancy-associated plasma protein-A [PAPP-A]) are closely associated with the risk of prostate cancer, especially playing an important role in risk stratification and early warning. The latest Mendelian randomization analyses show a positive correlation between serum IGF-I and prostate cancer risk (for example, in the UK Biobank data, each increase of one standard deviation in IGF-I correspondingly increases prostate cancer risk), and IGFBP-3 has also been confirmed as an important risk factor for prostate cancer and its progression (14, 19). Through the detection and dynamic monitoring of molecules such as IGF-I, it is expected that high-risk populations can be accurately identified before clinical symptoms appear, thus advancing the gateway of tumor prevention and control.

Notably, in most prospective and nested case-control studies included in this analysis, IGF-I concentrations were measured at baseline, years before prostate cancer diagnosis (7). This supports the early warning value of IGF-I.

#### 4.3.2 Precision screening and individualized risk stratification

IGF-I and its regulatory network can provide a molecular basis for prostate cancer screening and risk stratification, overcoming the limitations of specificity and sensitivity from relying solely on PSA. The latest multi-cohort meta-analyses and case-control studies indicate that IGF-I can not only distinguish prostate cancer patients from those with benign prostatic hyperplasia and normal individuals, but also further distinguish high Gleason grade (aggressive) tumors from indolent tumors (2, 15). Molecules such as PAPP-A, as key regulators of free IGF-I release, show unique value in tumor subtype stratification. The combined use of molecular and traditional indicators (PSA, family history, age, etc.) can optimize screening pathways and achieve risk stratification and precise management (15).

#### 4.3.3 Early prevention and health management interventions

Primary and secondary prevention of prostate cancer should focus on high-risk populations. The results of this study strengthen the theoretical basis for IGF-I as an early warning signal and support strengthening lifestyle interventions, regular follow-up, and health education in populations with elevated IGF-I. Existing epidemiological and intervention studies have shown that by adjusting dietary structure, increasing physical activity, and controlling body weight, it is possible to improve IGF-I and its downstream signaling and reduce the risk of tumor occurrence (2). At the public health level, the introduction of molecular markers helps improve screening efficiency, reduce resource waste and overdiagnosis, and promote the scientific and individualized development of prostate cancer prevention and control strategies (1).

#### 4.3.4 Advantages and limitations of IGF-I as a risk assessment tool

IGF-I and related molecules, as risk prediction tools, have certain advantages of independence and specificity, and can reflect individual biological susceptibility and changes in the tumor microenvironment. Especially among those with unclear family history or gray-zone PSA levels, IGF-I is expected to serve as a “bonus item” to improve the accuracy of risk assessment. However, IGF-I is influenced by multiple factors including genetics, environment, and detection methods, and its predictive performance shows significant heterogeneity among different populations and subtypes; a single molecular marker is difficult to fully reflect the complex carcinogenic process, and there is still a lack of globally recognized risk stratification thresholds and clinical decision-making standards (6–12, 14–16). Therefore, clinical application should advocate combined multi-index and multi-modal assessment, and risk models should be optimized according to the characteristics of different populations.

### 4.4 Limitation analysis

Despite our efforts to adhere to strict inclusion criteria and analytical standards, several important limitations remain that may affect the robustness and interpretation of our findings:

(1) Lack of Dose-Response Data

Although we confirmed a positive association between elevated serum IGF-I and increased prostate cancer risk, most included studies only compared the highest versus lowest IGF-I categories or used broad groupings. Detailed continuous data (such as per 10 ng/ml or per SD increase) were rarely reported, preventing a clear dose-response analysis. This makes it difficult to determine whether risk increases linearly or if there is a threshold effect.

(2) Heterogeneity and Influence of Study Characteristics

The included studies originated from diverse countries and populations, with significant variation in age, follow-up duration, and lifestyle, as well as study design (nested case-control, case-control, Mendelian randomization), detection methods, grouping criteria, and statistical approaches. Such heterogeneity likely contributes to the observed differences in effect estimates and may mask true associations. Although we attempted to explore these sources via subgroup and meta-regression analyses, no significant effect modifiers were identified, and the primary sources of heterogeneity remain unclear.

(3) Inconsistent Confounding Adjustment and Limited Control

Confounder adjustment varied widely: some studies only adjusted for basic demographic factors, while others considered additional variables such as family history, hormone levels, comorbidities, or lifestyle factors. The lack of original individual-level data precluded unified adjustment and restricted our ability to conduct stratified or sensitivity analyses. This may have led to residual confounding, causing the true association to be over- or underestimated.

(4) Unavailable Original Data

Our analysis was based entirely on published aggregate data. The absence of original raw data limited our capacity to perform more granular analyses (such as individual patient data meta-analysis or fine subgrouping) and to fully explore the dose-response relationship and interactions with other risk factors.

(5) Potential Publication Bias

Although funnel plots and statistical tests (Egger’s and Begg’s) suggested no major publication bias, we cannot exclude the possibility that studies with negative or null results were less likely to be published. Such bias could distort the pooled effect size.

In summary, while we applied multidimensional analyses and sensitivity tests to enhance reliability, our conclusions remain subject to limitations related to insufficient dose-response data, study heterogeneity, incomplete confounder adjustment, unavailable original data, and potential publication bias. Therefore, results should be interpreted with caution. Future research should prioritize multicenter, high-quality prospective studies, integration and sharing of individual-level data, and standardized reporting to provide more robust and comparable evidence.

### 4.5 Future research directions and prospects

Although our study suggests that serum IGF-I is associated with prostate cancer risk, more robust evidence is still required before clinical application. Future research should focus on the following aspects:

(1) Conduct Large-Scale, Multicenter Prospective Cohort Studies

To further validate the association between IGF-I and prostate cancer risk, future studies should prioritize well-designed, multicenter, large-sample prospective cohorts involving diverse populations and subgroups. Such studies will improve statistical power and enable a more precise evaluation of risk across different ethnic, age, and clinical backgrounds.

(2) Promote Integration and Sharing of Individual-Level Data

The integration of individual participant data (IPD) from multiple cohorts will facilitate unified covariate adjustment, enable comprehensive subgroup analyses, and allow for the robust assessment of dose-response relationships between IGF-I levels and cancer risk.

(3) Clarify Dose-Response Relationships and Address Confounding

Future studies should aim to collect and report detailed continuous IGF-I data, which would enable quantitative analysis of dose-response patterns. Comprehensive confounder assessment and advanced statistical modeling (e.g., multivariable adjustment, interaction analysis) are also essential to elucidate the true causal relationship and underlying biological mechanisms.

(4)Integrate Multi-Omics and Clinical Data

Combining IGF-I with multi-omics approaches (genomics, proteomics, metabolomics) and detailed clinical information will help to clarify the molecular mechanisms of IGF-I in prostate carcinogenesis and evaluate its utility for early screening, risk stratification, and individualized prevention strategies.

By addressing these directions, future research can enhance the scientific rigor and translational value of IGF-I as a biomarker, ultimately contributing to more precise prevention, early detection, and personalized management of prostate cancer.

### 4.6 The role of IGF-I in other malignancies

Epidemiological evidence has suggested a positive association between higher IGF-I levels and the risk of premenopausal breast cancer, as noted in previous large-scale prospective studies. The biological plausibility of these associations is supported by the established effects of IGF-I on cellular proliferation and inhibition of apoptosis. However, the magnitude and consistency of the association between IGF-I and cancer risk appear to differ according to cancer type, study design, and population characteristics. As discussed by Stattin et al., while IGF-I may act as a biomarker of susceptibility for multiple malignancies, further research is needed to clarify its clinical utility in risk prediction and prevention strategies across different cancer types (16).

## 5 Conclusion

This study evaluated the epidemiological evidence for serum insulin-like growth factor-1 (IGF-I) levels and the risk of prostate cancer, and the results indicate that high serum IGF-I levels are associated with an increased risk of prostate cancer. The combined effect value suggests that elevated IGF-I may be an independent risk factor for the occurrence of prostate cancer, but the dose-response relationship and consistency among different studies remain unclear. Subgroup and meta-regression analyses indicate that the association is influenced by multiple factors such as study population, detection methods, and confounding adjustment.

The results highlight the potential clinical application value of IGF-I and its regulatory molecules in prostate cancer risk assessment and early screening, which may help identify high-risk populations and achieve precise warning and stratified management. The existing evidence is still limited by study heterogeneity, insufficient control of confounding, and unclear dose relationships, and should be interpreted with caution.

In the future, it is necessary to strengthen multicenter, large-sample prospective studies and the integration of individual-level data, improve covariate adjustment and mechanism exploration, and promote the standardization and clinical translation of IGF-I-related indicators in the field of prostate cancer prevention and control, providing a more solid evidence base for optimizing disease screening strategies and health management.

## Data Availability

The original contributions presented in the study are included in the article/**Supplementary Material**. Further inquiries can be directed to the corresponding author.
