# Supplementary figures and images for: Serum insulin-like growth factor-1 and epidemiological evidence of the risk of prostate cancer
Source: Front Oncol. 2026 Jan 9;15:1730382. doi: 10.3389/fonc.2025.1730382 (PMC12827141; doi:10.3389/fonc.2025.1730382)

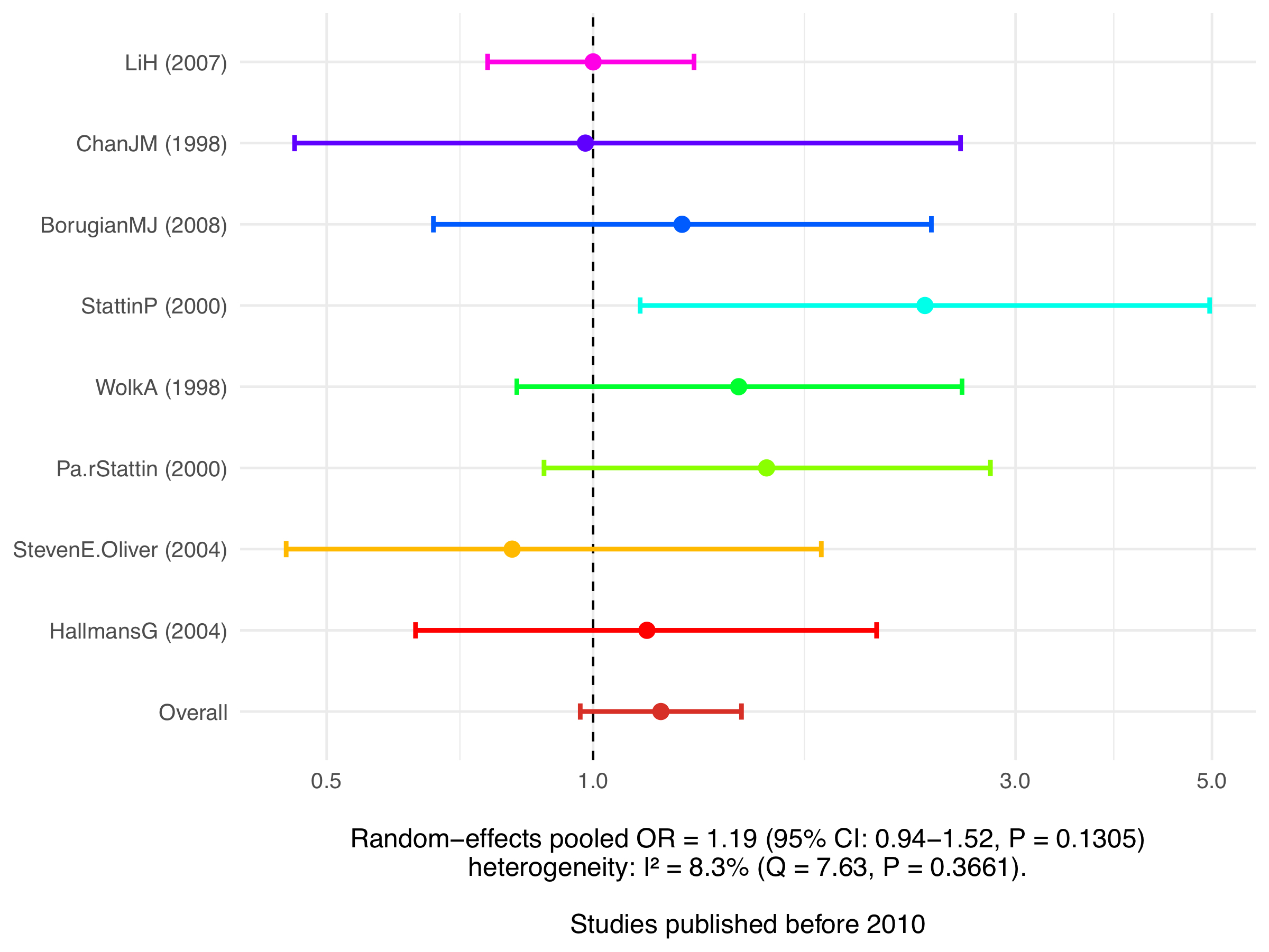

Supplement: Supplementary file 1 [file Image1.jpeg]

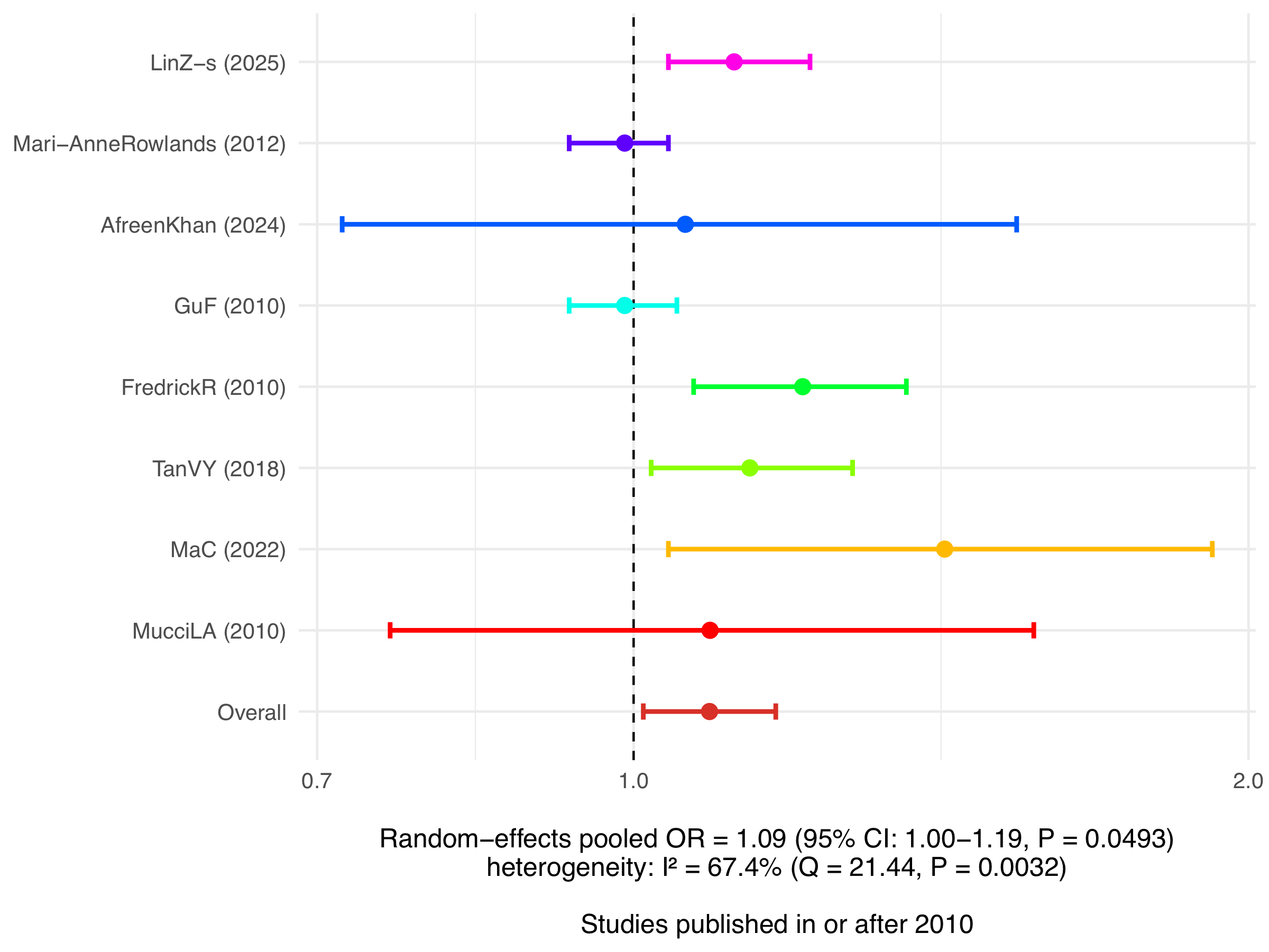

Supplement: Supplementary file 2 [file Image2.jpeg]

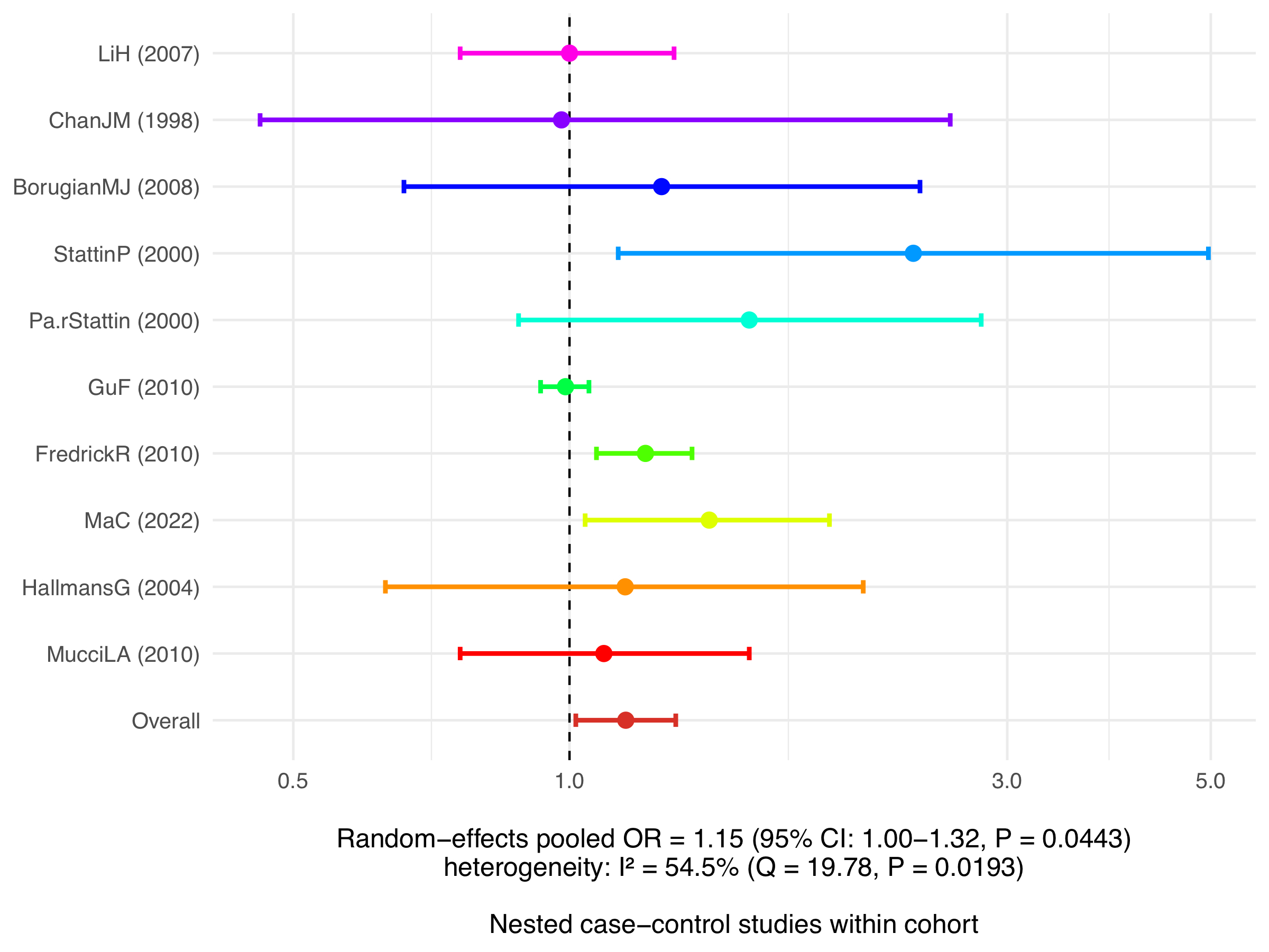

Supplement: Supplementary file 3 [file Image3.jpeg]

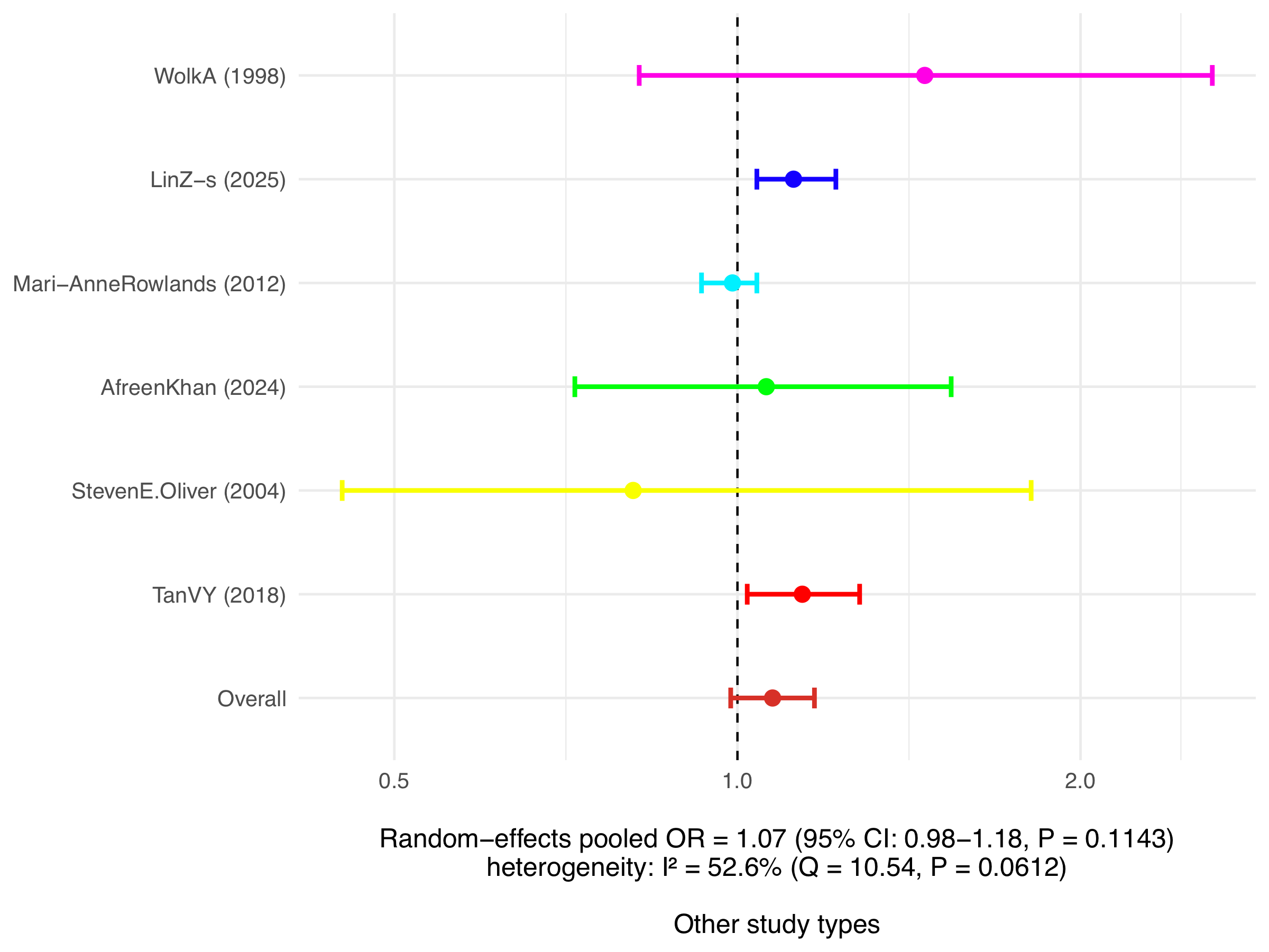

Supplement: Supplementary file 4 [file Image4.jpeg]

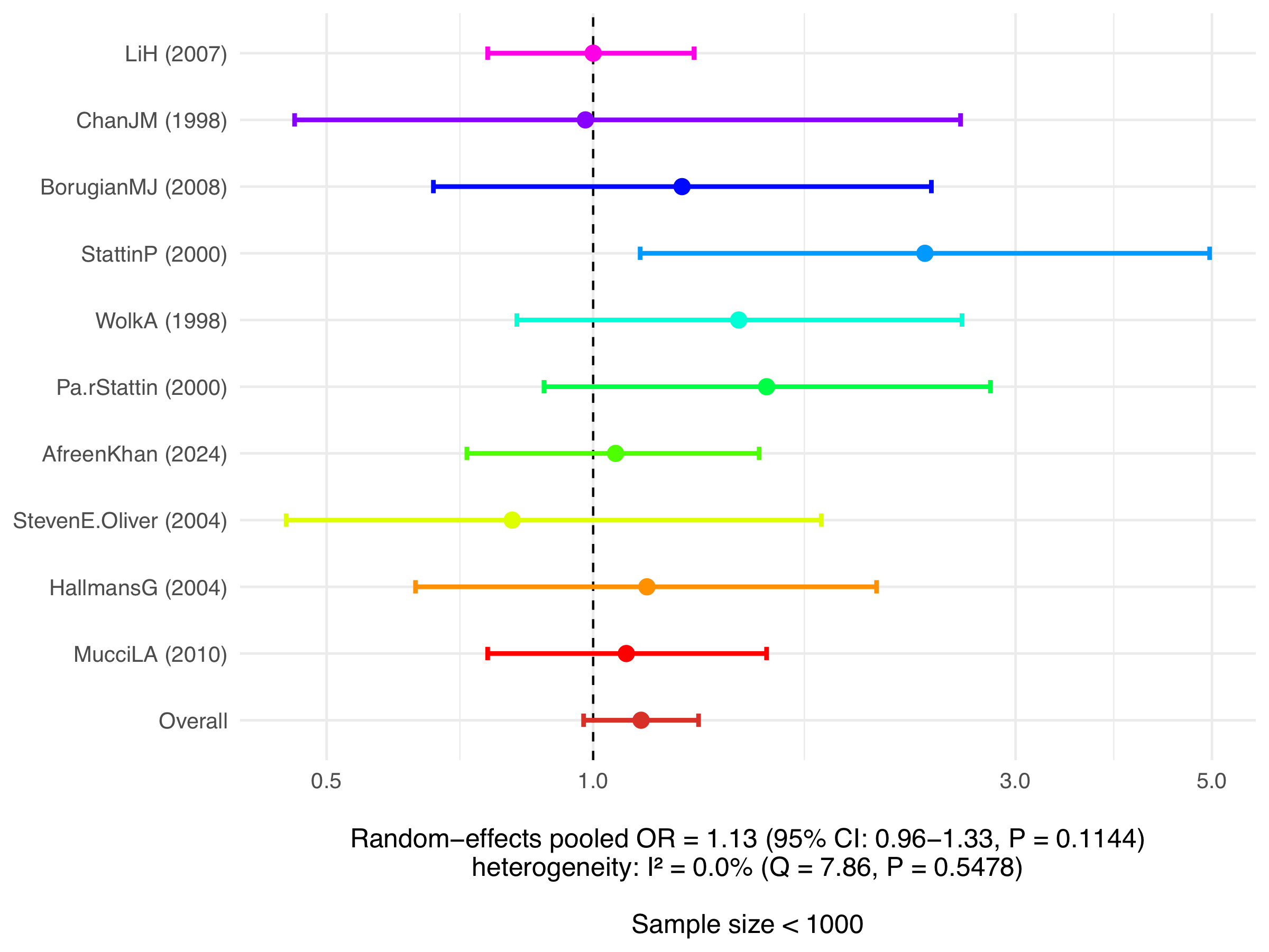

Supplement: Supplementary file 5 [file Image5.jpeg]

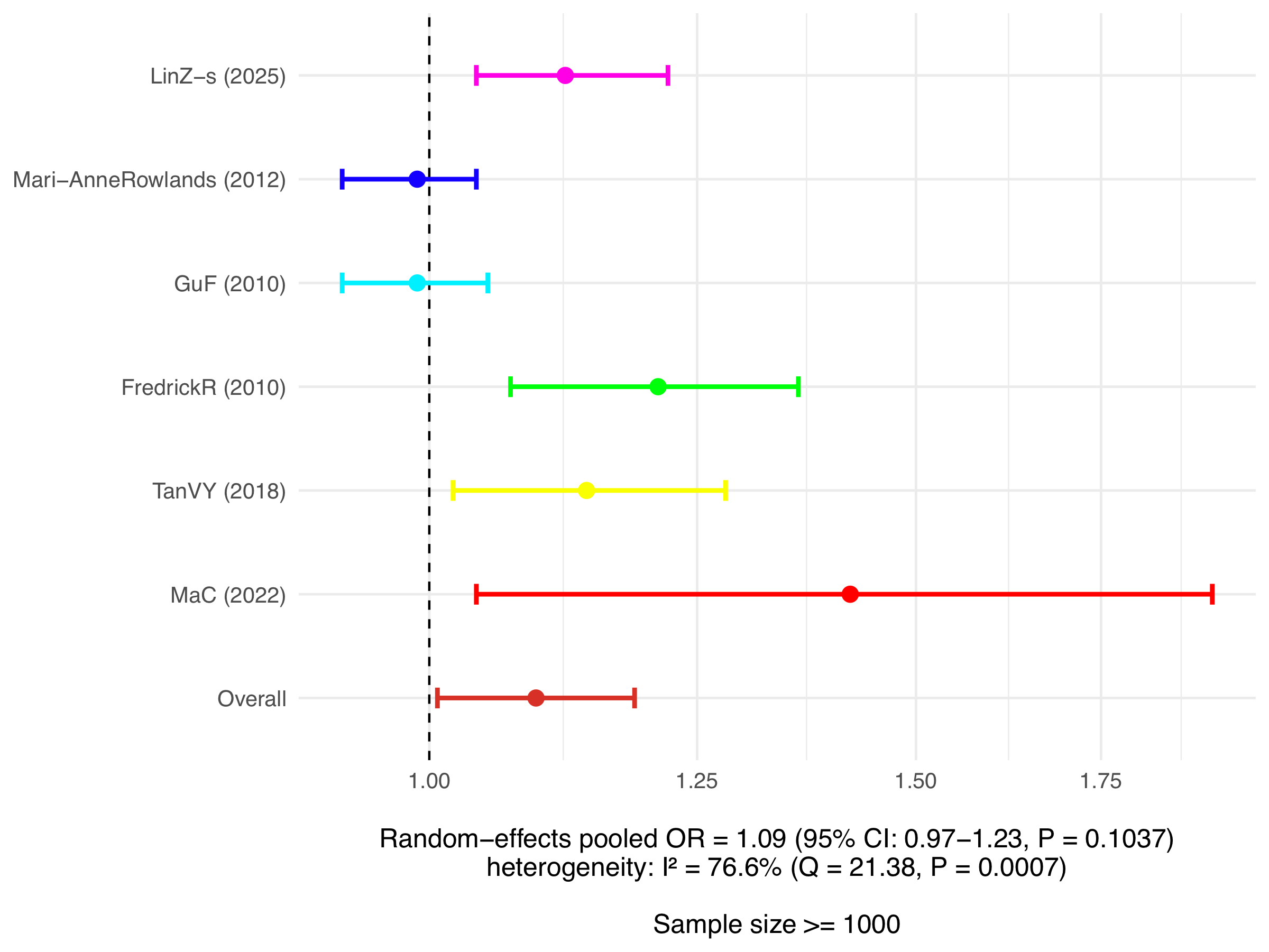

Supplement: Supplementary file 6 [file Image6.jpeg]
