# Supplementary material for: Serum insulin-like growth factor-1 and epidemiological evidence of the risk of prostate cancer
Source: Front Oncol. 2026 Jan 9;15:1730382. doi: 10.3389/fonc.2025.1730382 (PMC12827141; doi:10.3389/fonc.2025.1730382)
Supplement: Supplementary file 7 [file Table1.docx]

| Supplementary Table 1. Search strategy | | |
| --- | --- | --- |
| Database | Search strategy | Results |
| PubMed | ("Prostatic Neoplasms"[Mesh] OR "prostate cancer" OR "prostatic cancer" OR "prostate neoplasm*" OR "prostatic neoplasm*") AND("Insulin-Like Growth Factor I"[Mesh] OR "IGF-I" OR "IGF 1" OR "insulin like growth factor 1" OR "insulin-like growth factor-I")AND("Risk" OR "Incidence" OR "Epidemiology" OR "Cohort Studies"[Mesh] OR "Case-Control Studies"[Mesh] OR "case control" OR "cohort") | 461 |
| Embase | ('prostate cancer'/exp OR 'prostate cancer':ti,ab)AND('insulin like growth factor 1'/exp OR 'IGF-I':ti,ab)AND ('cohort study'/exp OR 'case control study'/exp)AND[humans]/limAND[english]/lim | 121 |
| Web of Science | ("prostate cancer" OR "prostatic cancer" OR "prostatic neoplasm*" OR "prostate neoplasm*")AND("insulin-like growth factor 1" OR "insulin like growth factor 1" OR "insulin-like growth factor-I" OR "IGF-I" OR "IGF 1")AND("risk" OR "incidence" OR "epidemiology" OR "case control" OR "cohort" OR "prospective" OR "retrospective") | 897 |
