# Supplementary material for: Serum insulin-like growth factor-1 and epidemiological evidence of the risk of prostate cancer
Source: Front Oncol. 2026 Jan 9;15:1730382. doi: 10.3389/fonc.2025.1730382 (PMC12827141; doi:10.3389/fonc.2025.1730382)
Supplement: Supplementary file 8 [file Table2.docx]

| Supplementary Table 2. Summary of Main Results | | | | | | | | | |
| --- | --- | --- | --- | --- | --- | --- | --- | --- | --- |
| First Author | Year | Region | Study Design | Sample Size | Age (years) | Follow-up (years) | Mean Serum IGF-I（ng/ml) | OR (95% CI) | Adjusted Factors |
| Li H | 2007 | US | NCC | 661 | 69.4±7.3 | 18 | 192.6 ± 2.9 | 1.00 (0.76 - 1.30) | Age, Smoking, Follow-up duration, IGFBP-3 |
| Chan JM | 1998 | US | NCC | 152 | 69.4±7.3 | 7 | 269.4 | 0.98 (0.46 - 2.60) | Age, Smoking, IGFBP-3, PSA, BMI, Height, Weight, Androgen, Carotenoids |
| Borugian MJ | 2008 | US/Canada | NCC | 96 | 67.1±6.1 | >1 | 236±75 | 1.26 (0.66 - 2.41) | Age, BMI |
| Stattin P | 2000 | Sweden | NCC | 149 | 67.4±7.2 | 6.7 | 194±62 | 2.37 (1.13 - 4.97) | Age, Smoking, BMI |
| Wolk A | 1998 | Sweden | CC | 210 | 50–74 | NA | 170.6 | 1.46 (0.82 - 2.61) | Age, BMI |
| Lin Z-s | 2025 | UK | MR | 291,274 | 57.1±7.7 | 10 | 134±37 | 1.12 (1.04 - 1.22) | Genetic instrumental variables, conventional confounders |
| Pär Stattin | 2000 | Sweden | NCC | 149 | 67.5±7.1 | 3.85 | 229 | 1.57 (0.88 - 2.81) | Age, Smoking, BMI, IGFBP-3 |
| Mari-Anne Rowlands | 2012 | UK | PBCC | 2,686 | 61.9±5.0 | NA | 163.5±54.9 | 0.99 (0.93 - 1.04) | Age, matched center, BMI, height, smoking, alcohol consumption, physical activity, socioeconomic status, family history, sample storage time, assay batch |
| Afreen Khan | 2024 | India | CC | 50 | 70.24±8.44 | NA | 360.47±27.03 | 1.06 (0.72 - 1.54) | Age, BMI, HDL, PSA, insulin, testosterone |
| Steven E. Oliver | 2004 | UK | PBCC | 176 | 62.2 | NA | 130.7 | 0.81 (0.45 - 1.81) | Age, center, blood draw date, smoking history, IGFBP-3 |
| Gu F | 2010 | Europe | NCC | 2,664 | 67.0±4.6 | 9.5 | 191.2 ± 77.6 | 0.99 (0.93 - 1.05) | Age, cohort, sex, batch |
| Fredrick R | 2010 | Europe | NCC | 6,012 | 68 | 10 | 153–176 | 1.21 (1.07 - 1.36) | Age, cohort, BMI, and other variables |
| Tan VY | 2018 | UK | MR | 44,825 | 62.59±5.00 | NA | 157.69±52.61 | 1.14 (1.02 - 1.28) | Age, BMI, smoking, diabetes |
| Ma C | 2022 | US | NCC | 1,302 | 68.5±7.1 | 15.7 | 143.0±34.2 | 1.42 (1.04 - 1.92) | Age, BMI, Gleason score, stage, cohort, blood collection time |
| Hallmans G | 2004 | Sweden | NCC | 149 | 61.7±6.6 | 2.6 | 156.3±45.7 | 1.15 (0.63 - 2.09) | Age, weight, height, smoking, diabetes, cholesterol |
| Mucci LA | 2010 | US | NCC | 545 | 68.6±6.2 | 8.5 | 117.3±31.7 | 1.09 (0.76 - 1.57) | Age, smoking, weight, diabetes, family history, blood draw time |
| Abbreviations:NCC, nested case-control study; CC, case-control study; MR, Mendelian randomization study; PBCC, population-based case-control study. | | | | | | | | | |
