# Supplementary material for: Serum insulin-like growth factor-1 and epidemiological evidence of the risk of prostate cancer
Source: Front Oncol. 2026 Jan 9;15:1730382. doi: 10.3389/fonc.2025.1730382 (PMC12827141; doi:10.3389/fonc.2025.1730382)
Supplement: Supplementary file 9 [file Table3.docx]

Supplementary Table 3

| Risk Of Bias In Non-randomized Studies of Interventions（ROBINS-I） | | | | | | | | |
| --- | --- | --- | --- | --- | --- | --- | --- | --- |
| Study | Bias due to confounding | Bias in selection of participants into the study | Bias in classification of interventions | Bias due to deviations from intended interventions | Bias due to missing data | Bias in measurement of outcomes | Bias in selection of the reported result | Overall risk of bias |
| Li H , 2007 | Moderate | Low | Low | Low | Low | Low | Low | Moderate |
| Chan JM , 1998 | Low | Low | Low | Low | Low | Low | Low | Low |
| Borugian MJ , 2008 | Moderate | Low | Low | Low | Low | Low | Low | Moderate |
| Stattin P , 2000 | Moderate | Low | Low | Low | Low | Low | Low | Moderate |
| Wolk A , 1998 | Moderate | Low | Low | Low | Low | Low | Low | Moderate |
| Pär Stattin , 2000 | Moderate | Low | Low | Low | Low | Low | Low | Moderate |
| Mari-Anne Rowlands , 2012 | Low | Low | Low | Low | Low | Low | Low | Low |
| Afreen Khan , 2024 | Moderate | Low | Low | Low | Low | Low | Low | Moderate |
| Steven E. Oliver , 2004 | Moderate | Low | Low | Low | Low | Low | Low | Moderate |
| Gu F , 2010 | Moderate | Low | Low | Low | Low | Low | Low | Moderate |
| Fredrick R , 2010 | Low | Low | Low | Low | Low | Low | Low | Low |
| Ma C , 2022 | Low | Low | Low | Low | Low | Low | Low | Low |
| Hallmans G , 2004 | Moderate | Low | Low | Low | Low | Low | Low | Moderate |
| Mucci LA , 2010 | Low | Low | Low | Low | Low | Low | Low | Low |

| Risk Of Bias in Mendelian Randomization Studies(ROB-MR) | | | | | | | | | | | |
| --- | --- | --- | --- | --- | --- | --- | --- | --- | --- | --- | --- |
| Study | Clear research question | Instrument relevance | Instrument independence | Exclusion restriction assumption | Pleiotropy assessment | Sample overlap | Population structure | Measurement error | Sensitivity analysis | Other (specify) | Overall risk of bias |
| Lin Z-s , 2025 | Low | Low | Moderate | Low | Low | Moderate | Moderate | Low | Low | Low | Moderate |
| Tan VY , 2018 | Low | Low | Low | Moderate | Moderate | Low | Moderate | Low | Moderate | Low | Moderate |
