# Supplementary material for: Serum insulin-like growth factor-1 and epidemiological evidence of the risk of prostate cancer
Source: Front Oncol. 2026 Jan 9;15:1730382. doi: 10.3389/fonc.2025.1730382 (PMC12827141; doi:10.3389/fonc.2025.1730382)
Supplement: Supplementary file 10 [file Table4.docx]

| Supplementary Table 4. GRADE Evidence Quality Assessment Table | |
| --- | --- |
| Assessment Dimension / Domain | Level/Description |
| Study Design | Prospective cohorts, case-control, Mendelian randomization |
| Initial Rating | Moderate |
| Consistency | Moderate to substantial heterogeneity; consistency limited in subgroups and meta-regression |
| Precision | Pooled effect 95%CI relatively narrow; sensitivity analysis robust |
| Directness | Most studies target population; clear definition of exposure and outcomes |
| Risk of Bias | Mostly low to moderate risk of bias; some studies with insufficient confounding adjustment |
| Publication Bias | No significant bias by Egger/Begg tests; potential underreporting of negative results |
| Factors for Upgrading | Inclusion of large, multicenter, prospective and genetic epidemiological studies |
| Factors for Downgrading | Considerable heterogeneity, residual confounding, reporting inconsistency, unclear dose-response |
| Overall Rating | Moderate |
